# Supplementary material for: Camel (Camelus spp.) Urine Bioactivity and Metabolome: A Systematic Review of Knowledge Gaps, Advances, and Directions for Future Research
Source: Int J Mol Sci. 2022 Nov 30;23(23):15024. doi: 10.3390/ijms232315024 (PMC9740287; doi:10.3390/ijms232315024)
Supplement: Supplementary file 1 [file ijms-23-15024-s001.zip › Supplementary Table S1.pdf]

Table S1. Results of Shapiro-Francia W' test with Box–Cox transformation.

Variable	Camel urine bioactivity					Camel urine metabolome				
	**Obs (n)**	**W'**	**V'**	**Z'**	**p-value**	**Obs (n)**	**W'**	**V'**	**Z'**	**p-value**
Journal	12	0.98190	0.337	-1.721	0.95741	8	0.98973	0.152	-2.442	0.99269
Year of publication	12	0.81277	3.489	2.204	0.01375	8	0.49848	7.439	3.778	0.00008
JCR Impact Factor per paper publication year	12	0.97777	0.414	-1.409	0.92055	8	0.79316	3.068	1.931	0.02674
Total number of citations per paper	12	0.83163	3.138	2.007	0.02238	8	0.76844	3.435	2.149	0.01580
Number of authors	12	0.93720	1.170	0.264	0.39595	8	0.99717	0.042	-3.673	0.99988
Country of corresponding author	12	0.96672	0.620	-0.778	0.78172	8	0.99487	0.076	-3.143	0.99917
Camel species	12	1.00000	0.000	-17.144	1.00000	8	0.99411	0.087	-3.011	0.99870
Camel breeding location	12	0.96558	0.641	-0.724	0.76554	8	0.98046	0.290	-1.704	0.95581
Sample size	5	0.87289	1.583	0.660	0.25457	4	0.0	0.0	0.0	0.0
Sex of sampled animals	12	1.00000	0.000	-18.128	1.00000	8	1.0000	0.000	-8.088	1.0000
Mean age of sampled animals (years)	5	1.0000	0.000	0.0	0.00001	2	0.0	0.0	0.0	0.0
Physiological status of sampled animals	12	0.99741	0.048	-4.404	0.99999	8	0.97918	0.309	-1.626	0.94802

A p-value less than 0.05 was considered statistically significant.

The W' value is the Shapiro–Francia test statistics. It is also reported the value V', which are more appealing indexes for departure from normality. The median values of V' are 1 for normal distributed samples, while

larger values mean nonnormality. The 95% critical values of this last statistics depend on the sample size and range between 1.2 and 2.4 (2.0 and 2.8); see Royston [1]. As one is just the transform of the other, neither V' nor W' contain different information from each other. The normal approximation to the sampling distribution of W' was done under the Box–Cox transformation, which is valid for $5 \leq n \leq 1000$.

1. Royston, P. Estimating departure from normality. Stat. Med. 1991, 10, 1283-1293.
